# Unilateral acute lung injury in pig: a promising animal model

**DOI:** 10.1186/s12967-022-03753-5

**Published:** 2022-11-26

**Authors:** Johannes Geilen, Matthias Kainz, Bernhard Zapletal, Silvana Geleff, Wilfried Wisser, Barbara Bohle, Thomas Schweiger, Marcus J. Schultz, Edda Tschernko

**Affiliations:** 1 Division of Cardiothoracic and Vascular Anesthesia & Critical Care Medicine, Department of Anesthesia, General Intensive Care and Pain Management, Medical University of Vienna, Waehringer Guertel 18-20, 1090 Vienna, Austria; 2 Department of Pathology, Medical University of Vienna, Vienna, Austria; 3 Department of Cardiac Surgery, Medical University of Vienna, Vienna, Austria; 4 Department of Pathophysiology and Allergy Research, Center of Pathophysiology, Infectiology and Immunology, Medical University of Vienna, Vienna, Austria; 5 Department of Thoracic Surgery, Medical University of Vienna, Vienna, Austria; 6 Department of Intensive Care, Academic Medical Center, University of Amsterdam, Amsterdam, The Netherlands

**Keywords:** Acute lung injury, Large animal model, Unilateral mechanical ventilation

## Abstract

**Background:**

Acute lung injury (ALI) occurs in 23% unilateral. Models of unilateral ALI were developed and used previously without clearly demonstrating the strictly unilateral nature and severity of lung injury by the key parameters characterizing ALI as defined by the American Thoracic Society (ATS). Thus, the use of unilateral ALI remained rare despite the innovative approach. Therefore, we developed a unilateral model of ALI and focused on the crucial parameters characterizing ALI. This model can serve for direct comparisons between the injured and intact lungs within single animals, thus, reducing the number of animals required for valid experimental conclusions.

**Methods:**

We established the model in nine pigs, followed by an evaluation of key parameters in six pigs (main study). Pigs were ventilated using an adapted left double-lumen tube for lung separation and two ventilators. ALI was induced in the left lung with cyclic rinsing (NaCl 0.9% + Triton® X-100), after which pigs were ventilated for different time spans to test for the timing of ALI onset. Ventilatory and metabolic parameters were evaluated, and bronchoalveolar lavage (BAL) was performed for measurements of inflammatory mediators. Finally, histopathological specimens were collected and examined in respect of characteristics defining the lung injury score (LIS) as suggested by the ATS.

**Results:**

After adjustments of the model (n = 9) we were able to induce strictly left unilateral ALI in all six pigs of the evaluation study. The median lung injury score was 0.72 (IQR 0.62–0.79) in the left lung vs 0.14 (IQR 0.14–0.16; p < 0.05) in the right lung, confirming unilateral ALI. A significant and sustained drop in pulmonary compliance (C_dyn_) of the left lung occurred immediately, whereas C_dyn_ of the right lung remained unchanged (p < 0.05). BAL fluid concentrations of interleukin-6 and -8 were increased in both lungs.

**Conclusions:**

We established a model of unilateral ALI in pigs, confirmed by histopathology, and typical changes in respiratory mechanics and an inflammatory response. This thoroughly evaluated model could serve as a basis for future studies and for comparing pathophysiological and pharmacological changes in the uninjured and injured lung within the same animal.

**Supplementary Information:**

The online version contains supplementary material available at 10.1186/s12967-022-03753-5.

## Background

Acute respiratory distress syndrome (ARDS) is the most common pulmonary complication in critically ill patients that often results in unfavorable outcomes. Approximately two out of five patients with ARDS die [1], and the recent COVID-19 pandemic taught us that outcome of patients with ARDS due to infectious causes has not changed over recent years [2, 3], despite significant improvements in ventilatory management and various other aspects of care for critically ill ARDS patients.

Clinical research in ARDS patients remains extremely difficult, not in the last place, because critically ill patients are a heterogeneous population with a variety of causes for acute lung injury [4]. Therefore, animal models are needed. Indeed, several animal models have provided important insights into the pathogenesis and potential treatments for ARDS, leading to changes in care in patients suffering from this condition [5, 6].

Acute lung injury (ALI) is rarely evenly distributed in both lungs. Recently findings of the large LUNG SAFE study demonstrated that ALI develops unilateral in approximately 23% of ARDS patients [7, 8], e.g., after unilateral chest trauma [9], unilateral aspiration [10], or with unilateral pneumonia [11]. Models of unilateral ALI have been used to study ventilator induced lung injury [12–14]. However, none of these models evaluated the specific parameters demanded by the American Thoracic Society (ATS) to characterize ALI. Results of experimental procedures could vary substantially if various degrees of lung destruction are present. Therefore, our group has developed a strictly unilateral model of acute lung injury in mechanically ventilated pigs.

Here, we describe a pioneering model of unilateral acute lung injury, induced by cyclic rinsing with 0.9% NaCl and Triton® X-100 in mechanically ventilated pigs. We fully characterized lung injury, focusing on the technique of inducing lung injury, macroscopic and microscopic changes, respiratory mechanics and hemodynamics, and inflammatory markers as defined by the ATS.

## Methods

Animal experiments were performed at the animal facilities of the Center of Biomedical Research of the Medical University of Vienna. The study was conducted after approval from the Ethics Committee for Animal Research and the permission of the Federal Ministry of Education, Science and Research according to §26 TVG 2012 (Ethics Protocol Number: 162/115/97/98). To ensure optimal animal handling prior to the experiments, pigs were housed in groups, kept on dry straw beds and fed in a manner appropriate for their species.

**Fig. 1:**
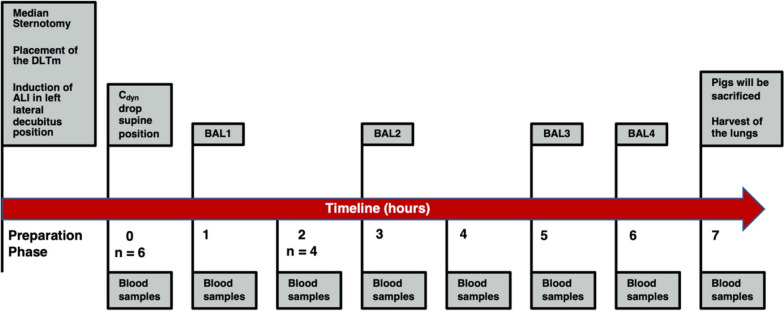
Overview of timeline, interventions and sampling periods. Timepoint 0 marks the beginning of measurements after induction of unilateral ALI. Two animals were sacrificed after 1 h. DLTm: modified double-lumen tube; C_dyn_: dynamic compliance; BAL: bronchoalveolar lavage

The study was structured in two parts: (a) development of unilateral ALI (n = 9) and (b) systemic evaluation of key parameters defining ALI (n = 6), which was regarded as the main study. Figure 1 depicts the main study design and timeline of the experimental interventions.

### Preparation of a modified double lumen tube

**Fig. 2:**
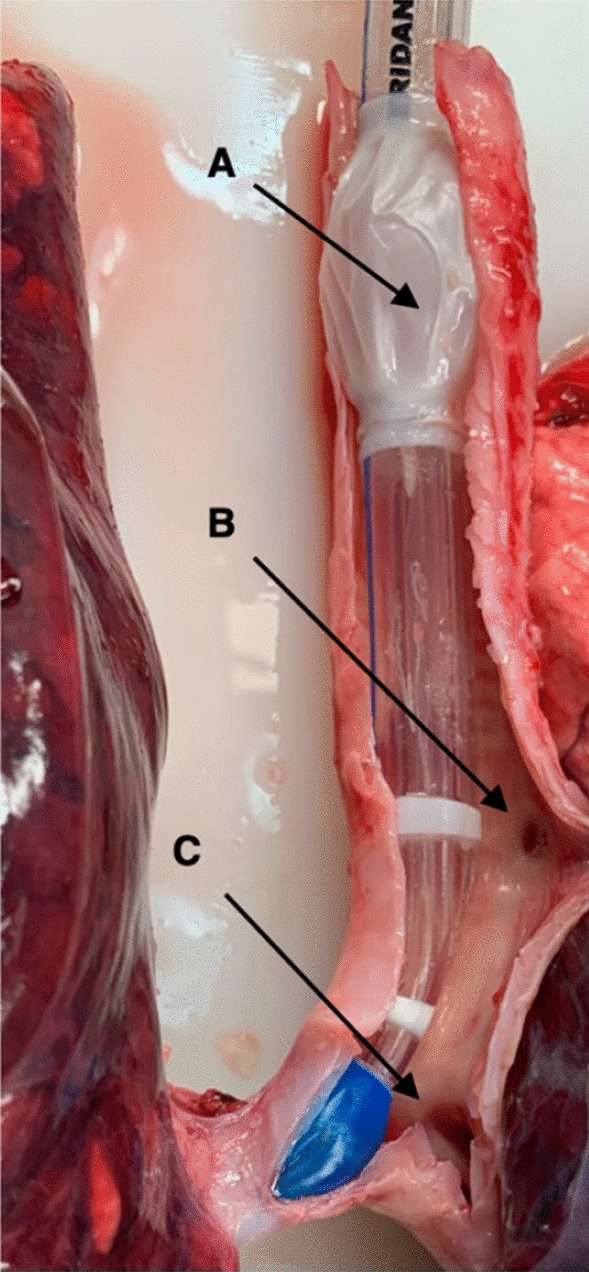
DLTm in situ at the end of the experiment, dorsal view. **A** Shows the proximally everted tracheal cuff to prevent blockage of the bronchus trachealis (**B**). The tip of the DLTm sits in the left main bronchus, distal to the carina (**C**)

In pigs, a tracheal bronchus branch, named the bronchus trachealis, originates from the trachea proximal to the carina to aerate the upper parts of the right lung (Fig. 2) [15]. To fit the anatomy of the bronchial tree of pigs, we modified a commercial left-sided double-lumen tube for left bronchial intubation in humans (37Fr, Hudson RCI®, Sheridan® Sher-i-bronch®, Teleflex Medical, Morrisville, NC, USA). This adaption was mandatory to avoid blockage of the bronchus trachealis so that ventilation of the right upper lobe was guaranteed. Use of a commercially available left double-lumen tube would have resulted in atelectasis of the right upper lobe. For this, the tracheal cuff was detached from the double lumen tube at the distal end and everted 4 cm proximally. The cuff was fixed and glued (Silastic®, Medical Adhesive Silicon Type A, Dow Corning Corporation, Midland, MI, USA) to the tube in the proximal position (Additional file 1: Supplement Fig. S1). The cuff was then partially inflated with 2 to 3 mL of air to avoid undesirable adherence of the cuff material to the tube in the adjunct areas of the fixation zone. After a drying period of at least 72 h the cuff was tested for air leakage with a cuff pressure of 30 cmH_2_O. If no cuff pressure drop was observed within 5 min the cuff was deemed to be airtight, and the double lumen tube could be used in the pig experiments.

### Animal preparation

All pigs were premedicated with ketamine 5–6 mg kg^−1^ i.m., midazolam 0.2–0.3 mg kg^−1^ i.m. and medetomidine 0.05–0.1 mg kg^−1^ i.m. and placed in the supine position on a heating mat. After cannulation of an ear vein, propofol 2.5–5 mg kg^−1^ i.v. and 100 µg fentanyl i.v. was administered after which the pig was orotracheally intubated with a commercial singlelumen endotracheal tube (Rüschelit® Super Safety Clear, Teleflex Medical, Co. Westmeath, Ireland) with an ID of 8.0 mm. Pigs were ventilated using a human ventilator (Dräger Primus®, Dräger Medical, Luebeck, Germany) using a volume-controlled mode with tidal volumes of 6–8 mL kg^−1^ and positive end-expiratory pressure (PEEP) of 5 cmH_2_O, resulting in maximum inspiratory pressures (P_max_) of ~ 20 cmH_2_O, at a respiratory rate of 12–20 breaths per minute. Anesthesia was maintained with continuous infusion of 2% propofol (20 mg kg^−1^ h^−1^), fentanyl (5 µg kg^−1^ h^−1^) and rocuronium (1 mg kg^−1^ h^−1^). A central venous catheter was placed in the external jugular vein under ultrasound guidance, and an arterial line was placed in the left or right common carotid artery. A suprapubic urinary catheter (Zystofix®, B. Braun SE, Melsungen, Germany) was placed for urinary output measurements under ultrasound guidance. After instrumentation and preoxygenation of the pigs, the commercial single lumen tube was replaced by the modified double-lumen tube (DLTm) over a tube exchange catheter (Cook Medical, Limerick, Ireland). The position of the double-lumen tube was bronchoscopically verified (Pentax® FI-13RBS, Pentax Europe GmbH, Hamburg, Germany) in all animals. The position of the tube was adjusted until the tracheal bronchus, the carina and the left and right main stem bronchus were confirmed. After correct positioning of the double-lumen tube, the left and right lung were ventilated separately using two ventilators, one ventilator for ventilation of the right lung and another ventilator for ventilation of the left lung (Additional file 1: Supplement Fig. S2). For both lungs, we used volume-controlled mode with tidal volumes of 3 mL kg^−1^ for the left lung and 5 mL kg^−1^ for the right lung. A positive end-expiratory pressure (PEEP) of 5 cmH_2_O, resulted in maximum inspiratory pressures (P_max_) of ~ 20–25 cmH_2_O in the right lung and ~ 15–20 cmH_2_O in the left lung, at a respiratory rate of 12–20 breaths per minute. Then, animals underwent a median sternotomy, and bilateral pleural opening was performed to have free access to the right and the left lung. Lung separation was followed by temporary closure of the thorax with clamps and hypoxic preconditioning of the left lung. Hypoxic preconditioning was performed by transient termination of ventilation of the left lung while ventilating the right lung with 100% oxygen for 10 min. Hypoxic preconditioning was performed to account for the Euler-Liljestrand mechanism [16] and, thus, support hemodynamic stability and to minimize shunt fraction during induction of lung injury. After 10 min of hypoxic preconditioning, pigs underwent three cycles of rinsing of the left lung with 0.9% NaCl and Triton® X-100. Animals in the pilot study underwent rinsing in the supine position, whereas animals in the main study were positioned in the left lateral decubitus position before cyclic rinsing. The correct tube position was reconfirmed via fiberoptic bronchoscopy in all animals of the pilot study and the main study.

### Pilot study

A pilot study with nine pigs was conducted as a first step to achieve strictly unilateral ALI in pigs weighing 49–64 kg. This included the optimal production of the DLTm. We evaluated the optimal position of the tracheal cuff to avoid atelectasis of the upper parts of the right lung. In three pigs, the lung block was harvested with the DLTm tube in place, and the distance between the everted tracheal cuff and the tracheal bronchus was measured. We found that a proximal shift of the tracheal cuff of 4 cm was sufficient to avoid blockage of the tracheal bronchus.

Despite optimal positioning of the tracheal cuff and optimal blockage of the left main bronchus with the DLTm spillover of the rinsing fluid occurred in 6 out of 9 pigs, thus injuring parts of the right lung. After unsuccessful production of unilateral conditions of lung injury in 6 out of 9 pigs we decided to perform the rinsing procedure in the left lateral decubitus position. This step proved to be essential in producing strictly unilateral ALI.

### Main study

Six male Austrian Landrace pigs (Sus scrofa domesticus) with an average body weight of 62 kg (range 56 to 76 kg) were used for the experiments. Cyclic rinsing of the left lung was performed in all pigs of the main study in the left lateral decubitus position. Otherwise, the experiments were conducted in a manner comparable to the pilot study.

### Induction of unilateral lung injury

Lung injury was induced in all animals of the pilot study and the main study by rinsing the left lung with a mixture of 150 mL 0.9% saline and 0.3% Triton® X-100 (Sigma Aldrich, Saint Louis, MO, USA). Rinsing was performed in the supine position in all animals of the pilot study and in the left lateral decubitus position in all animals of the main study. During the purging of the left lung, continuous bronchoscopic control via the tracheal lumen was used to detect any spill-over of the rinsing solution into the right lung in all animals of the pilot study and the main study. These measures were undertaken to guarantee strict unilateral rinsing. To ensure optimal distribution of the rinsing fluid in the peripheral lung tissue, the left lung was ventilated with increasing P_max_ of up to 40 cmH_2_O for 5 min. Thereafter, the remaining rinsing liquid in the left bronchial system was removed via suction. This rinsing procedure was repeated twice; thus, 3 cycles of rinsing were performed in all pigs. A mean arterial pressure > 70 mmHg was set as the target to guarantee hemodynamic stability during the vulnerable rinsing period. Boluses of phenylephrine (Biorphen®, Sintetica, Muenster, Germany) 0.2 mg were administered, if required.

### Ventilation

After induction of lung injury, ventilation of the right lung was adjusted to reach the predefined target values for respiratory and metabolic stability, wherein we targeted a PaO_2_ > 60 mmHg, a PaCO_2_ < 65 mmHg, and SpO_2_ > 90%. The left lung was ventilated using lung-protective ventilation with V_T_ of 3 mL kg^−1^, PEEP of 8 cmH_2_O and Pmax of < 30 cmH_2_O. In cases of desaturations or hypercapnia, ventilatory settings were adjusted. Eight out of nine pigs were ventilated for 7 h in the pilot study (one animal died prematurely). Two pigs were ventilated for one hour, and four pigs were ventilated for seven hours in the main study.

### Lung lavage and tissue sampling

BAL was performed in all pigs (n = 9) of the pilot study and three pigs of the main study for measurement of interleukins (IL-6 and IL-8). All of these animals underwent mechanical ventilation for at least 7 h. No BAL was performed in animals with ventilation time ≤ 1 h. One animal died prematurely and was therefore excluded from the analysis of IL-6 and IL-8 in BAL. Bronchoalveolar lavage of the left and right lung was carried out at fixed time intervals (at 1, 3, 5, and 6 h) to measure typical inflammatory parameters, such as interleukin-6 (IL-6) and interleukin-8 (IL-8). BAL in the left and right lung was performed with a saline bronchial flush of 40 mL saline 0.9%, which was sampled again via suction. Samples were stored immediately on ice and were centrifuged at 2000×*g* at 4 °C for 10 min, and shock frozen at − 80 °C within 3–5 min thereafter. For analysis, samples were thawed and centrifuged at 1000×*g* for 20 min. Cytokine levels were determined using commercial ELISA kits for IL-6 (MyBioSource, San Diego, CA) and IL-8 (Thermo Fisher Scientific, Waltham, MA).

At the end of the experiment, pigs were sacrificed with an overdose of fentanyl 500 µg i.v. and 30 mL potassium chloride 7.45% i.v.. Thereafter, the lungs were removed en-bloc. Six lung tissue samples were obtained from standardized areas in the left and right lung (Additional file 1: Supplement Fig. S3), thus, 12 tissue samples were collected from each pig. Lung samples were processed and analyzed by an independent and blinded pathologist to test for histological signs of inflammation and destruction. Then, 7.5% buffered paraformaldehyde was used to fix the lung tissue for at least 12 h. Fixed lung tissue was then cut to size, placed in tissue capsules, drained overnight, and embedded in paraffin the next morning. Sections of two micrometers thickness were cut and stained with hematoxylin–eosin or with trichrome staining for better visualization of fibrin (pink) and erythrocytes (orange) using acid fuchsin orange G aniline blue (according to Mallory & Cason). The tissue samples were stored in paraformaldehyde at 4 °C until evaluated and graded according to the Lung Injury Scoring System (LISS) adapted from Matute-Bello et al*.*, and used previously by other authors for numerical evaluation of lung injury [17].

### Statistical analysis

For the main study we defined LISS as main outcome parameter, because histopathological changes are rated crucial in defining ALI in the consensus paper of the American Thoracic Society [17]. A LISS of zero is associated with healthy lung structure and a LISS of one (1.0) is associated with completely destroyed lung structure. The precondition of strictly unilateral ALI is a substantial difference in LISS between the right and the left lung. Therefore, we demanded a mean difference in LISS between the right and left lung of 0.5 and assumed a rather large standard deviation in differences of 0.2. With alpha of 0.05 a power of 0.9 can be achieved with six pigs in paired samples. Thus, a sample size of six pigs was chosen for the main study.

Because of the small sample size Wilcoxon-signed-rank-test was used for comparison of outcome parameters throughout. Descriptive statistics were expressed as mean ± SD, the significance level was set at p ≤ 0.05. IBM SPSS Statistics software (Version 27.0.1.0) was used for data processing.

## Results

To test for functional parameters defining ALI, histological evaluation, functional damage to lung tissue and inflammation were assessed. Nine pigs were included in the pilot study for developing and establishing the model, and six pigs were included in the main study. In the pilot study, unilateral ALI was finally achieved by performing cyclic rinsing in the left lateral decubitus position. One pig out of nine died prematurely in the pilot study. In the main study, the reproducibility of this new model was tested. All animals of the main study (n = 6) completed the experiments, and no animal died prematurely.

Conclusions gained in the pilot study regarding the successful construction of the modified double-lumen tube (DLTm) and the correct positioning of the animals during cyclic rinsing are shown in the methods section. All six animals in the main study were examined with respect to changes in ventilatory mechanics and histopathological signs of lung injury. Three of six pigs underwent repeated bronchoalveolar lavage (BAL) cycles for collection of BAL specimens and subsequent analysis of IL-6 and IL-8. BAL was not performed in the other three animals. Additionally, IL-6 and IL-8 were analyzed in the BAL fluid of eight pigs from the pilot study. Therefore, we present IL-6 and IL-8 from a total of 11 pigs.

**Fig. 3:**
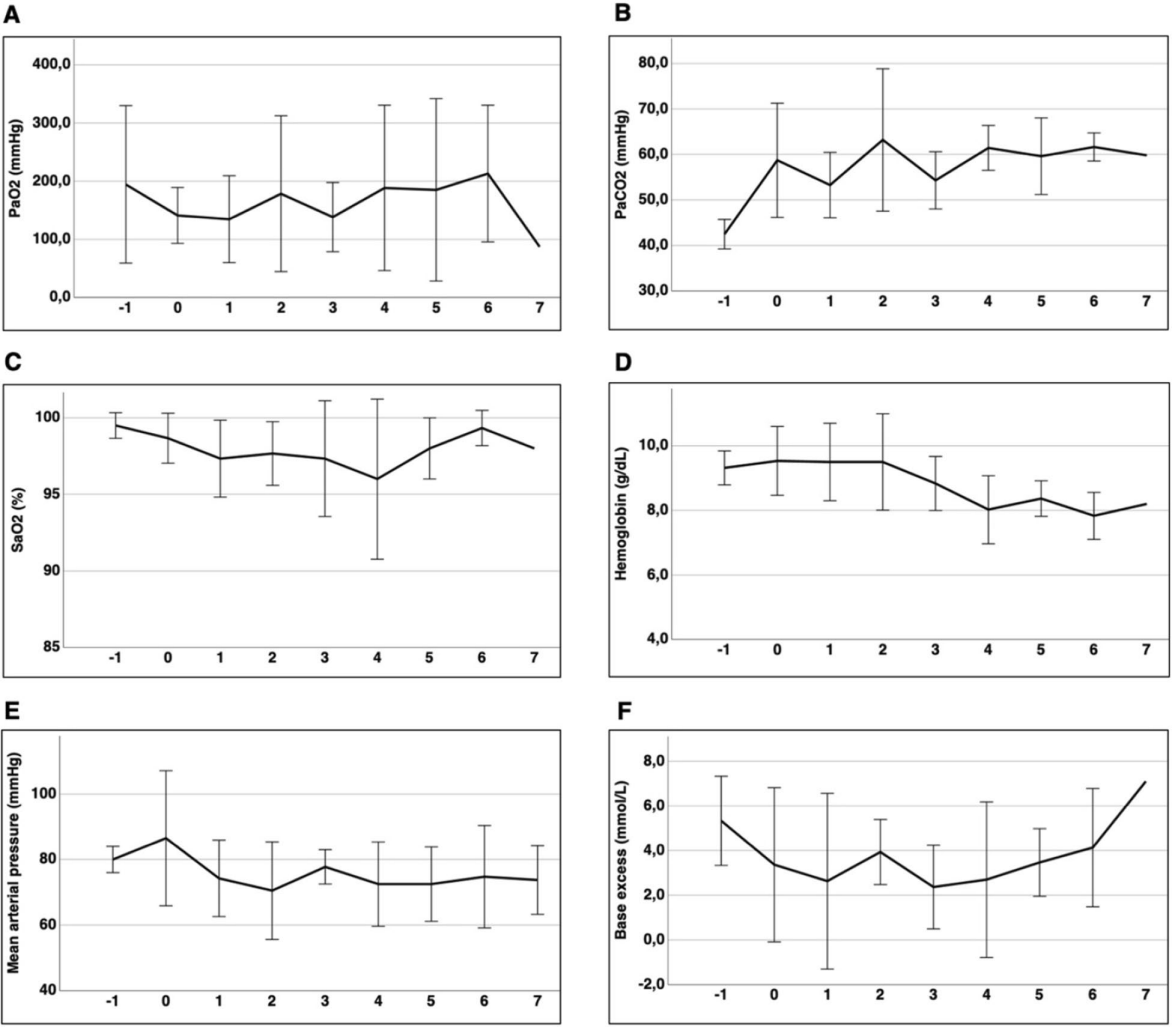
Representation of hemodynamics and metabolics during unilateral ALI. Data are shown as the mean ± SD; time in hours; timepoint − 1 = baseline measurements; timepoint 0 = immediately after ALI induction; time in hours. **a**–**c** Sum of respiratory data of p_a_O_2_, p_a_CO_2_ and S_a_O_2_. **d** hemoglobin concentration. **e** Mean arterial pressure and **f** base excess over time

The pigs had comparable baseline hemodynamics, ventilatory parameters and metabolic parameters. Additionally, hemodynamic, metabolic and ventilatory settings as defined previously could be achieved in all animals throughout the experiment (Fig. 3 and Additional file 1: Supplement Table S1). Parameters defining ventilation are shown in detail in Table 1.

**Table 1:** Respiratory parameters after ALI induction (left and right are separated; main study)

	Immediately after ALI (0 h)		1 h after ALI		2 h after ALI		3 h after ALI		4 h after ALI		5 h after ALI		6 h after ALI		7 h after ALI	
	Left	Right	Left	Right	Left	Right	Left	Right	Left	Right	Left	Right	Left	Right	Left	Right
Compliance (C_dyn_)	6.7 ± 2.1^†^ (n = 5)	18.3 ± 2.3 (n = 5)	5.8 ± 2.4^†^ (n = 6)	15.9 ± 4.3 (n = 6)	6 ± 2.4 (n = 4)	15.3 ± 2.6 (n = 4)	6.3 ± 2.6 (n = 4)	14.9 ± 1.5 (n = 4)	6.2 ± 2.5 (n = 3)	15.4 ± 4.8 (n = 3)	5.7 ± 3.9 (n = 3)	13.3 ± 4 (n = 3)	4.8 ± 3.4 (n = 4)	11.8 ± 2.7 (n = 4)	4.8 ± 3.4 (n = 4)	12.4 ± 3.4 (n = 4)
FiO_2_ (%)	0.4 ± 0.12 (n = 5)	0.38 ± 0.08 (n = 5)	0.49 ± 0.19 (n = 4)	0.49 ± 0.23 (n = 4)	0.44 ± 0.18 (n = 4)	0.46 ± 0.23 (n = 4)	0.44 ± 0.18 (n = 4)	0.44 ± 0.18 (n = 4)	0.35 ± 0.05 (n = 3)	0.35 ± 0.05 (n = 3)	0.4 ± 0.1 (n = 3)	0.45 ± 0.13 (n = 3)	0.48 ± 0.17 (n = 4)	0.5 ± 0.18 (n = 4)	0.47 ± 0.21 (n = 3)	0.55 ± 0.18 (n = 3)
Tidal volume (VT)	141 ± 42^†^ (n = 5)	353 ± 60 (n = 5)	155 ± 54 (n = 4)	350 ± 22 (n = 4)	156 ± 41 (n = 4)	333 ± 21 (n = 4)	149 ± 28 (n = 4)	348 ± 18 (n = 4)	149 ± 47 (n = 3)	349 ± 12 (n = 3)	129 ± 82 (n = 3)	333 ± 16 (n = 3)	124 ± 71 (n = 4)	327 ± 18 (n = 4)	105 ± 92 (n = 3)	321 ± 5 (n = 3)
etCO_2_ (mmHg)	12.3 ± 7.1 (n = 4)	57.4 ± 12.2 (n = 5)	26.5 ± 30.4 (n = 2)	52.3 ± 3.2 (n = 3)	32 ± 19.8 (n = 2)	56.3 ± 5.5 (n = 3)	32.5 ± 27.6 (n = 2)	55 ± 8.5 (n = 3)	33 ± 36.8 (n = 2)	53.3 ± 11.9 (n = 3)	24.7 ± 24.1 (n = 3)	59 ± 5.3 (n = 3)	24.3 ± 24.2 (n = 4)	58.7 ± 10 (n = 3)	22 ± 28.3 (n = 2)	58.5 ± 13.4 (n = 2)
PEEP (cmH_2_O)	7 ± 2.2 (n = 5)	5.4 ± 0.6 (n = 5)	6.8 ± 2.5 (n = 4)	5.8 ± 0.5 (n = 4)	8 ± 0 (n = 4)	5.8 ± 0.5 (n = 4)	8 ± 0 (n = 4)	5.8 ± 0.5 (n = 4)	8 ± 0 (n = 3)	5.7 ± 0.6 (n = 3)	8 ± 0 (n = 3)	5.7 ± 0.6 (n = 3)	8 ± 0 (n = 4)	5.8 ± 0.5 (n = 4)	8 ± 0 (n = 3)	5.7 ± 0.6 (n = 3)
Ppeak (cmH_2_O)	30.5 ± 7.1 (n = 4)	30 ± 6.7 (n = 4)	32.7 ± 2.1 (n = 3)	26.3 ± 3.1 (n = 3)	34 ± 5.3 (n = 3)	28.3 ± 3.1 (n = 3)	34 ± 5.3 (n = 3)	29.3 ± 2.9 (n = 3)	32 ± 0 (n = 2)	29 ± 2.8 (n = 2)	32.5 ± 0.7 (n = 2)	30 ± 2.8 (n = 2)	35.3 ± 4.9 (n = 3)	31.7 ± 3.5 (n = 3)	37.5 ± 6.4 (n = 2)	32 ± 4.2 (n = 2)
Pplat (cmH_2_O)	29.6 ± 7.3 (n = 4)	27.5 ± 7.7 (n = 4)	32.7 ± 2.1 (n = 3)	25.3 ± 4.7 (n = 3)	33.7 ± 5.7 (n = 3)	27.7 ± 4.2 (n = 3)	34 ± 5.3 (n = 3)	28.7 ± 4 (n = 3)	32 ± 0 (n = 2)	27.5 ± 5 (n = 2)	32 ± 0 (n = 2)	28.5 ± 5 (n = 2)	35 ± 5.2 (n = 3)	30.7 ± 5.1 (n = 3)	37 ± 7.1 (n = 2)	30.5 ± 6.4 (n = 2)
Respiratory rate (RR)	21.8 ± 3.9 (n = 4)	27.3 ± 14.2 (n = 4)	20.7 ± 2.3 (n = 3)	25.3 ± 4.2 (n = 3)	20.7 ± 2.3 (n = 3)	25.3 ± 4.2 (n = 3)	21.3 ± 3.1 (n = 3)	24.7 ± 1.2 (n = 3)	22 ± 5.7 (n = 2)	24 ± 2.8 (n = 2)	22 ± 5.7 (n = 2)	27.5 ± 3.5 (n = 2)	23.3 ± 2.3 (n = 3)	24.7 ± 5 (n = 3)	24 ± 2 (n = 3)	24.7 ± 5 (n = 3)
I:E	1:1.3 ± 0.1 (n = 3)	1:1.5 ± 0.1 (n = 3)	1:1.2 (n = 1)	1:1.4 (n = 1)	1:1.2 (n = 1)	1:1.4 (n = 1)	1:1.2 (n = 1)	1:1.5 (n = 1)	1:1.2 (n = 1)	1:1.5 (n = 1)	1:1.2 (n = 1)	1:1.5 (n = 1)	1:1.2 (n = 1)	1:2 (n = 1)	1:1.2 (n = 1)	1:2 (n = 1)

Values are expressed as mean ± SD

FiO_2_: fraction of inspired Oxygen; PEEP: positive end-expiratory pressure; I:E: inspiratory time/expiratory time ratio; Ppeak: peak ventilatory pressure; Pplat: plateau pressure

^†^p < 0.05

### Histopathological proof of lung injury

**Fig. 4:**
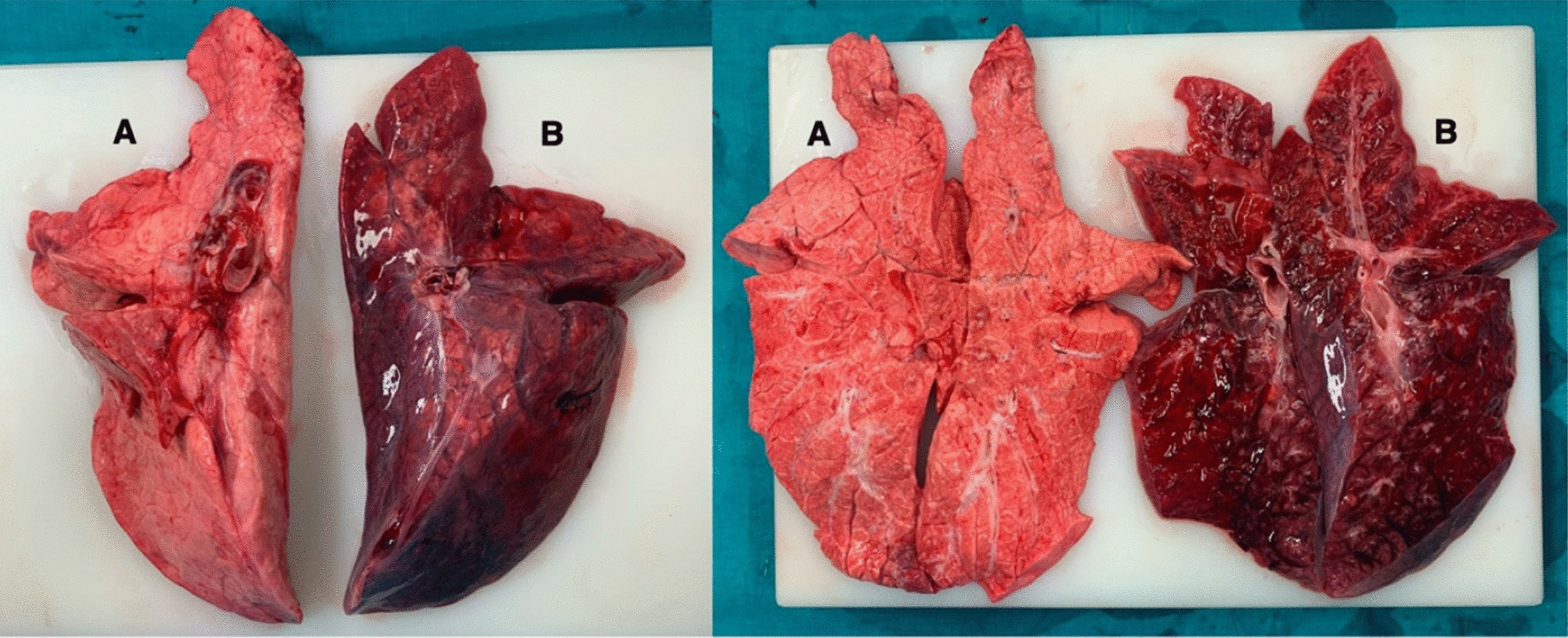
Macroscopic result at the end of the experiment. **A** Represents the right lung without visible sights of ALI, **B** shows the damaged left ALI lung. ALI induction occurred in left lateral decubitus position

Macroscopic signs of lung damage of the left lung were present in all pigs in the pilot study (n = 9) as well as in the main study (n = 6). The right lung showed no macroscopically visible signs of damage in all animals of the main study (Fig. 4), whereas six out of nine animals showed macroscopic signs of right sided lung damage in the pilot study group due to spill-over (Additional file 1: Supplement Fig. S4). Strict unilateral left lung damage occurred in all pigs of the main study independent of the elapsed time between ALI induction and harvesting of the lungs. Comparable macroscopic results were achieved in pigs ventilated for one hour and for seven hours after ALI induction.

**Fig. 5:**
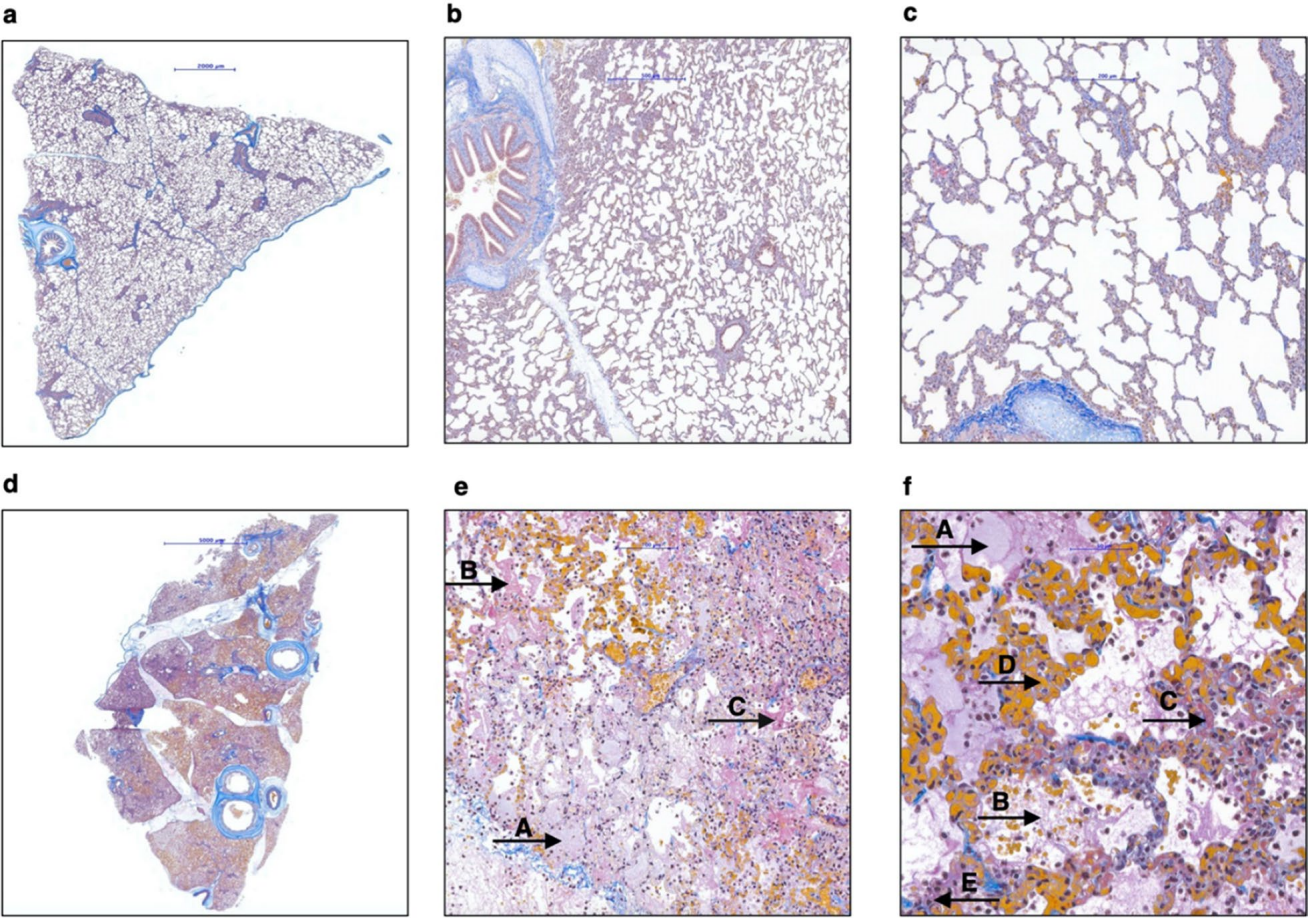
Microscopic differences in healthy lung (**a**–**c**) versus ALI lung (**d**–**f**); SFOG coloring. **a**–**c** Specimens derived from the right lung show a normal lung architecture with thin alveolar septa and cell-free alveoli (marked scale 2000 µm, 500 µm and 200 µm, respectively); **d** overview of a specimen derived from the left lung (marked scale 5000 µm); **e**, **f** edema in the alveoli (**A**), proteinaceous debris-fibrin (**B**), hyaline membranes (**C**), hemorrhage and congestion of the capillaries (**D**) and neutrophilic infiltration (**E**) can be observed (marked scale 100 µm and 50 µm)

**Fig. 6:**
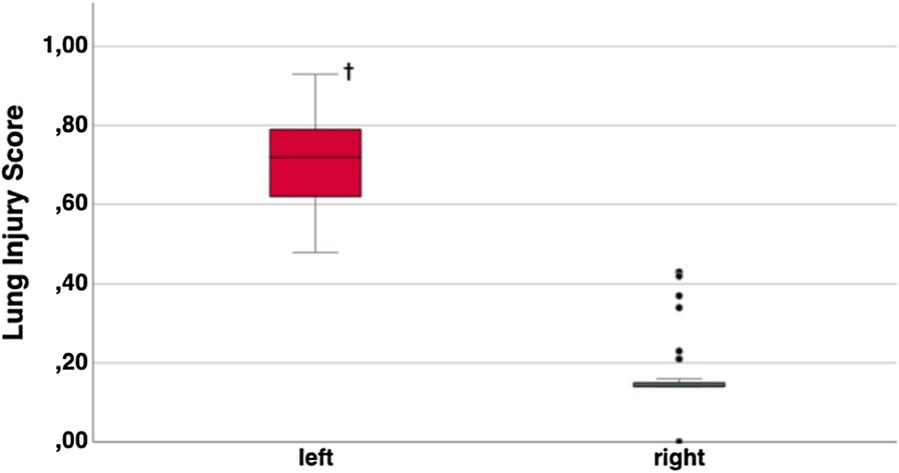
Adapted Lung Injury Scoring System (LISS). Healthy lung (right) compared to ALI lung (left). Thirty-six specimens were analyzed for the left and right sides, for a total of 72 specimens. ^†^p < 0.05

Histopathological findings were in line with the macroscopic examination of the lungs. Histopathological analysis showed typical signs of ALI [17] in the left lungs whereas the normal histological structure was preserved in the right lungs (Fig. 5). To ensure a reliable grading of the lung damage, the adapted Lung Injury Scoring System (LISS, Additional file 1: Supplement Table S2) was used for evaluation of all histological samples in the main experiment: 12 specimens per pig, 72 specimens in all six pigs in total. The score ranges from 0 (no detectable lung damage) to 1.0 (maximum destruction of lung tissue). The median score of the histological samples of the left lung was 0.72 (IQR 0.62–0.79), and the median score of the histological samples of the right lung was 0.14 (IQR 0.14–0.16), p = 0.031 (Fig. 6).

### Signs of functional damage

**Fig. 7:**
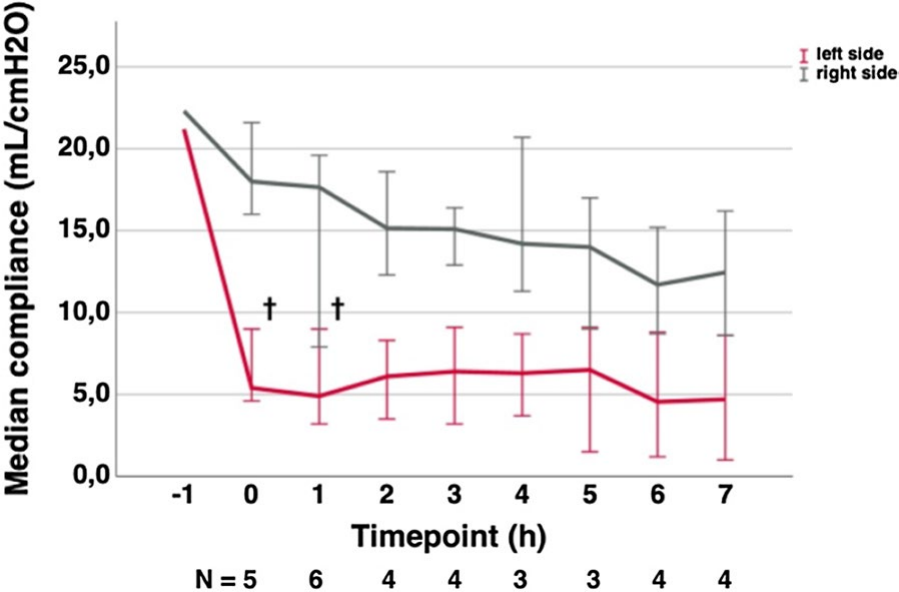
Dynamic compliance. Dynamic compliance (mL/cmH_2_O) over time in hours (error bars 95% CI). Timepoint − 1 = baseline measurements; timepoint 0 = immediately after ALI induction; time in hours. ^†^p < 0.05

A severely impaired gas exchange could not be observed in the arterial blood gas analyses, due to the preserved gas exchange in the right lung targeting at an S_p_O_2_ > 90%. Dynamic lung compliance (C_dyn_) was evaluated since it is severely impaired in ALI. Baseline C_dyn_ was comparable between the left lung and right lung in all animals (C_dyn_ > 20 mL cmH_2_O^−1^). A drop in C_dyn_ < 10 mL cmH_2_O^−1^ occurred in the left lung immediately after ALI induction. This significant decrease persisted over the entire course of the experiment (p = 0.031; Fig. 7). A slight decrease in compliance occurred in the right lung over time, probably due to the high ventilatory pressures required to preserve stable metabolic values.

### Detection of inflammation in BAL fluid

**Fig. 8:**
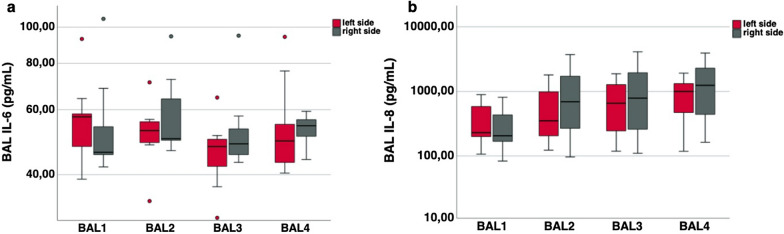
Interleukin levels (pg/mL) in BALF. Levels in the left lung (red) versus right lung (gray) 1 h (BAL1), 3 h (BAL2), 5 h (BAL3) and 6 h (BAL4) after ALI induction. **a** Depicts IL-6 levels, and **b** depicts IL-8 levels, respectively

The values for IL-6 in BAL are depicted in Fig. 8a (n = 11). Four consecutive BAL cycles were performed 1 h, 3 h, 5 h and 6 h after ALI induction. Elevated levels of IL-6 were observed in the BAL specimens of the left lung but also in the specimens derived from the right lung. In BAL1, one hour after ALI induction, the median IL-6 level on the left side was 57.4 pg/mL (IQR 47.8–58.4 pg/mL) versus 46.0 pg/mL (IQR 45.4–54.1 pg/mL) on the right side. IL-6 levels in BAL2 averaged 52.7 pg/mL (IQR 48.9–55.7 pg/mL) on the left side compared to 50.1 pg/mL (IQR 49.6–64.2 pg/mL). In BAL3 IL-6 levels were 48.5 pg/mL (IQR 45.4–53.3 pg/mL) in contrast to 47.7 pg/mL (IQR 42.4–49.9 pg/mL) in the BAL of the left lung. BAL4 exhibited median IL-6 levels of 54.3 pg/mL (IQR 50.9–56.4 pg/mL) in the BALs of the right lung compared to median IL-6 levels of 49.4 pg/mL (IQR 43.2–54.8 pg/mL) in the BALs of the left lung. Overall, there was no significant difference in IL-6 levels in BAL between the left and right lungs (p = 0.365).

Furthermore, IL-8 levels were analyzed at comparable time intervals, as shown in Fig. 8b (n = 11). A steady increase in IL-8 occurred over time on both sides. In BAL1, the median IL-8 level in the left lung was 229.7 pg/mL (IQR 200.9–586.7 pg/mL) compared to 206.1 pg/mL (IQR 170.7–437.1 pg/mL) on the right side. The median IL-8 level in BAL2 of the left lung was 351.2 pg/mL (IQR 206.3–1026.0 pg/mL) compared to 694.3 pg/mL (IQR 269.2–1735.3 pg/mL) in BALs derived from the right lungs. The median IL-8 level in BAL3 was 659.3 pg/mL (IQR 253.2–1303.2 pg/mL) on the left side versus a median IL-8 level of 787.3 pg/mL (IQR 345.1–2170.6 pg/mL) in BAL on the right side. In BAL4 of the left lung, the median IL-8 values were 1001.4 pg/mL (IQR 493.0–1326.3 pg/mL) and 1247.7 pg/mL (IQR 477.6–2340.6 pg/mL) in BALs derived from right lungs. In summary, there was no significant side difference in IL-8 levels detected in BALs from the right lungs in comparison to IL-8 levels detected in BALs from the left lungs (p = 0.206).

We examined the 6 pigs with strict unilateral ALI separately (3 pigs from the pilot study, 3 pigs from the main study) and were not able to determine any significant difference as well (Wilcoxon-signed-rank-test for IL-6: p = 0.563 and for IL-8: p = 0.156).

## Discussion

Our main goal was to create a model of strictly unilateral ALI, which exhibits all major criteria of ALI described in the consensus statement of the American Thoracic Society [17]: ALI had to be present in the left (rinsed) lung, whereas the right (unrinsed) lung of the animal had to remain intact. Especially advantageous about this model is that the animal serves as its own control enabling paired statistical testing, associated with reduced numbers of animals required while still achieving statistical power. Numerous experimental studies examining pulmonary pathophysiology and drug effects are likely to benefit from this model. A lavage model using warm saline 0.9% in combination with a detergent (Triton® X-100) was chosen to induce unilateral ALI. Animal models using lipopolysaccharide (LPS) injection or cecal ligation and puncture for indirect ALI induction [18] were not suitable to generate unilateral conditions. After lavage, the left lung was ventilated with injurious peak pressures of up to 40 mbar, further contributing to lung damage (two hit model). The major difference to existing models of unilateral ALI [12–14] is the proof for strictly unilateral development of ALI. All key parameters, i.e. histological proof, typical changes in ventilatory mechanics, and inflammatory parameters were present in our model pinpointing the unilateral character of ALI.

We assessed the main features of ALI in the left and right lung to test for unilateral conditions: histological evidence, measurements of physiological dysfunction and measurements of the inflammatory response. Unilateral ALI was induced in a reproducible manner in all six animals.

### Histopathology

Typical histological evidence of ALI assesses neutrophilic infiltration of the interstitium and the alveolar space, formation of hyaline membranes and edema of the alveolar walls. These histological findings are summarized in the lung injury score initially introduced by Matute-Bello et al. [17] Most investigators use an adapted form of the score [18–20]. Since the evidence of hemorrhage is regarded as a somewhat relevant feature we included it in the adapted histological score. The left lung showed macroscopically severe destruction, whereas the right lung proved to be macroscopically unchanged in our model. The adapted histological score showed significant evidence of the typical signs of ALI in the left lung and normal values for the right lung. Therefore, histological evaluation proved the unilateral character of ALI in our model.

Since the animals were sacrificed approximately eight hours after induction of ALI, the typical features of the early, exudative phase of ALI were pronounced, whereas fibroblastic remodeling and collagen deposition in the lung associated with the fibrotic phase were not found [21–23]. This is typical for the majority of animal models [17] since the exudative phase of ALI lasts approximately 7 days after induction of ALI [24].

### Functional impairment

Generally arterial oxygen tension (P_a_O_2_) and P_a_O_2_/F_i_O_2_ are used to prove functional impairment and determine the severity of ARDS in humans (the Berlin definition for ARDS) [25]. Due to the unilateral character of our model, P_a_O_2_ and the P_a_O_2_/F_i_O_2_ ratio remain in a physiological range, and thus, are unsuitable parameters for the evaluation of functional impairment in our model. Since the right and left lungs were ventilated with two separate ventilators, dynamic compliance (C_dyn_) served as a parameter indicating the onset of severe lung injury. C_dyn_ dropped within 15 min after cyclic rinsing below 50% of baseline in the left lung and remained in the normal range in the right lung. The drop in compliance indicated rapid functional deterioration of the left lung.

The preservation of P_a_O_2_ and metabolic values in our animals is an advantage over bilateral models because the incidence of premature death before finishing the experiment was extremely low in our study when compared to recent reports of bilateral ALI in pig models [26]. Repeated lung lavage without detergence is also associated with hemodynamic and metabolic stability but does not lead to severe epithelial damage or nor to a substantial reduction in C_dyn_ [27], making the model unsuitable for studies investigating strategies of protective ventilation. Models known to reproduce epithelial injury seen in human ARDS/ALI, such as the oleic acid model or the endotoxin model, lead to severe structural damage but cannot be used in a unilateral model. Additionally they are associated with severe hemodynamic and metabolic instability [27]. Therefore, the combination of metabolic and hemodynamic stability in combination with severe histologically proven epithelial damage and an immediate and sustained drop in C_dyn_, as observed in our unilateral ALI model, seems to be of advantage.

An immediate decrease in C_dyn_ is also associated with models using tracheal application of gastric juice or hydrochloric acid (HCl) [28]. Lung injury exhibits a patchy pattern in models using HCl or gastric juice. The patchy pattern can be misleading if small regions of the lung are used to study drug effects, etc. In addition, the amount of lavage fluid administered also seems to be linked to the severity and time course of onset of ALI. Meers et al. used 4 mL/kg (150 mL) for ALI induction in a bilateral ALI model. In our model, 3–4 mL/kg saline 0.9% + Triton® X-100 0.3% was instilled solely in the left lung, and the rinsing was repeated twice. This might, in addition to the use of detergent, explain the more rapid drop in C_dyn_ in our model compared to the model of Meers et al. In contrast, the porcine ALI model of Tiba et al. was initiated with no more than 1 mL/kg gastric juice producing only mild ALI after 12 h [29].

### Inflammation

Another hallmark in ALI models is the measurement of cytokines reflecting the degree of inflammation. Meduri et al. showed that high cytokine levels (TNFalpha, IL-1β and IL-8) during the exudative phase of ARDS negatively affect the severity of ARDS and mortality in humans [30]. In the early period of ARDS alveolar macrophages are activated by proinflammatory chemokines. As a result, toxic mediators are released and lead to a further increase in the inflammatory cascade [21]. The systemic proinflammatory cytokines IL-6 and IL-8 are associated with adverse outcomes in ARDS [31]. Therefore, we assessed IL-6 and IL-8 in our study.

We found similar or even higher interleukin levels in BAL compared to other animal models of ALI [26, 28, 32–34]. Additionally, the amount of released mediators correlates with the severity and onset of ALI. In a novel model of ALI induction with smoke inhalation [26], IL-6 levels increased no earlier than 48 h after smoke inhalation. In contrast, we observed an increase in IL-6 in BAL as early as 1 h after lung lavage. Additionally, the concentrations measured in our model were 3–4 times higher than those observed by Leiphrakpam et al. [26], indicating a massive inflammatory reaction in our unilateral animal model. In line with this, we also found increased IL-8 levels in BAL. IL-8 levels increased further during the course of the experiment. This time course of IL-8 release is in line with other findings [21, 35].

### Possible benefits of unilateral ALI for future toxicological and pharmacological studies: mitochondria targeting and antioxidants

We detected severely elevated IL-6 and IL-8 levels in the healthy and the injured ALI lung suggesting severe systemic inflammation due to development of unilateral ALI in our animals. Elevation of interleukins was observed as well in the histopathological healthy right lung. Pro-inflammatory mediators cause oxidative stress and lead consecutively to severe damage and death of cells. Oxidative stress can also be a side effect of various drugs. Antioxidants are of enormous research interest to protect against or ameliorate oxidative stress [36–38] caused by inflammation or drug toxicity. Since our model can be regarded as a model of inflammation and/or toxic injury it offers a potent possibility to test for the protective effects of antioxidants of various kinds.

Various environmental compounds, drugs and xenobiotics induce their deleterious side effects thorough increased reactive oxygen species (ROS) production [39]. Mitochondria and lysosomes were shown to be primary targets for toxicity induced increased membrane permeability associated with the release of free radicals [38, 40]. Our animal model could serve for testing of protective effects of many ROS scavenger agents in healthy and already severely injured lung tissue.

### Limitations

All animal models have that try to mimic human ARDS. However, pig animal models have been reported to show better translational potential due to similarities in anatomy and physiology [41]. Lung injury in ARDS has been reported to develop in three pathophysiological phases: the exudative phase, the proliferative phase, and the fibrotic phase [22]. Since our animals were sacrificed within 8 h after induction of ALI, it can be representative only for the exudative phase of the disease.

Our model does not appear to be able to determine any left-to-right difference in IL-6 and IL-8 despite successfully induced unilateral ALI. Although the levels of the interleukins are much higher than physiological normal values, the progression is more or less parallel when comparing the left and right lung. It can be assumed that the massive inflammation in the left lung leads to a systemically relevant inflammatory reaction and as a result, interleukins are also released in epithelial lining fluid of the right, healthy lung while its function and gas exchange are preserved.

Another potential downside of our unilateral pig model is the technical complexity of inducing unilateral ALI. The adaptions of the commercially available double-lumen tube for humans are not very demanding. However, the bronchoscopic placement of the tube requires an experienced physician because it is key in producing unilateral ALI and avoiding spill-over of lavage fluid to the healthy lung. Additionally, the changes in position (lateral decubitus to supine) require close attention to hemodynamic stability and expert use of vasopressors (norepinephrine and phenylephrine). Furthermore, the experimental animals should weigh between 45 and 65 kg to achieve a correct and stable tube position. Therefore this protocol is unsuitable for piglets.

## Conclusion

In conclusion, we have succeeded in establishing a new ALI model for unilateral ALI in pigs, in which only the left lung is specifically affected and meets all the currently applicable requirements for a successfully induced ALI. Pronounced histopathological changes in the lung parenchyma and a severe functional limitation of the left lung were demonstrated with normal lung tissue and intact function of the right lung. In addition, there were massively increased interleukin-6 and -8 levels in BAL fluid. The unilateral ALI model in pigs can be used to answer various research questions requiring comparison of damaged and healthy lung tissue.

## Supplementary Information

**Additional file 1: Supplement Figure S1.** Modification of the left-sided double-lumen tube (DLT). (**A**) presents a standard 37Fr Hudson RCI®, Sheridan® Sher-i-bronch® for left main bronchus intubation. (**B**) shows the modified DLT where the tracheal cuff is shifted 4 cm proximal in comparison with the standard tube. **Supplement Figure S2.** Separation of ventilation of the right lung and the left lung. (**a**) depicts the DLTm in situ with one ventilator connected to the tracheal lumen (white) and one ventilator connected to the bronchial lumen (blue). (**b**) depicts the ventilators performing ventilation of the left lung and ventilation of the right lung. **Supplement Figure S3.** Lung zones for histopathological analysis. Schematic representation of the six zones of tissue sampling after the experiment; upper lobe, mid field and lower lobe, each ventral and dorsal, respectively. **Supplement Figure S4.** Macroscopic preparation of a spill-over lung in the pilot study. Successful generation of ALI in the left lung (**L**) but clearly visible spill-over and injury in the lower lobe of the right lung (**R**). ALI induction occurred in supine position. **Supplement Table S1.** Hemodynamics, metabolics and oxygenation before and after ALI induction over time (main study). **Supplement Table S2.** Lung injury score (adapted from Matute-Bello et al. [17]).

## Data Availability

The datasets used and/or analysed during the current study are available from the corresponding author on reasonable request.
