# Supplementary material for: Unilateral acute lung injury in pig: a promising animal model
Source: J Transl Med. 2022 Nov 26;20:548. doi: 10.1186/s12967-022-03753-5 (PMC9701381; doi:10.1186/s12967-022-03753-5)
Supplement: Supplementary file 1 — Additional file 1: Supplement Figure S1. Modification of the left-sided double-lumen tube (DLT). (A) presents a standard 37Fr Hudson RCI®, Sheridan® Sher-i-bronch® for left main bronchus intubation. (B) shows the modified DLT where the tracheal cuff is shifted 4 cm proximal in comparison with the standard tube. Supplement Figure S2. Separation of ventilation of the right lung and the left lung. (a) depicts the DLTm in situ with one ventilator connected to the tracheal lumen (white) and one ventilator connected to the bronchial lumen (blue). (b) depicts the ventilators performing ventilation of the left lung and ventilation of the right lung. Supplement Figure S3. Lung zones for histopathological analysis. Schematic representation of the six zones of tissue sampling after the experiment; upper lobe, mid field and lower lobe, each ventral and dorsal, respectively. Supplement Figure S4. Macroscopic preparation of a spill-over lung in the pilot study. Successful generation of ALI in the left lung (L) but clearly visible spill-over and injury in the lower lobe of the right lung (R). ALI induction occurred in supine position. Supplement Table S1. Hemodynamics, metabolics and oxygenation before and after ALI induction over time (main study). Supplement Table S2. Lung injury score (adapted from Matute-Bello et al. [17]). [file 12967_2022_3753_MOESM1_ESM.docx]

**SUPPLEMENT**

**
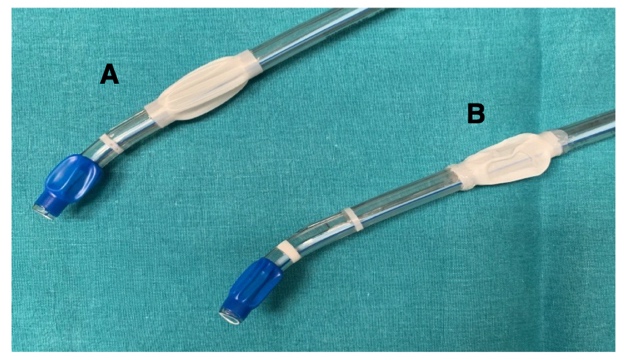
**

**Figure S1: Modification of the left-sided double-lumen tube (DLT)**

(**A**) presents a standard 37Fr Hudson RCI^®^, Sheridan^®^ Sher-i-bronch^®^ for left main bronchus intubation. (**B**) shows the modified DLT where the tracheal cuff is shifted 4 cm proximal in comparison with the standard tube.


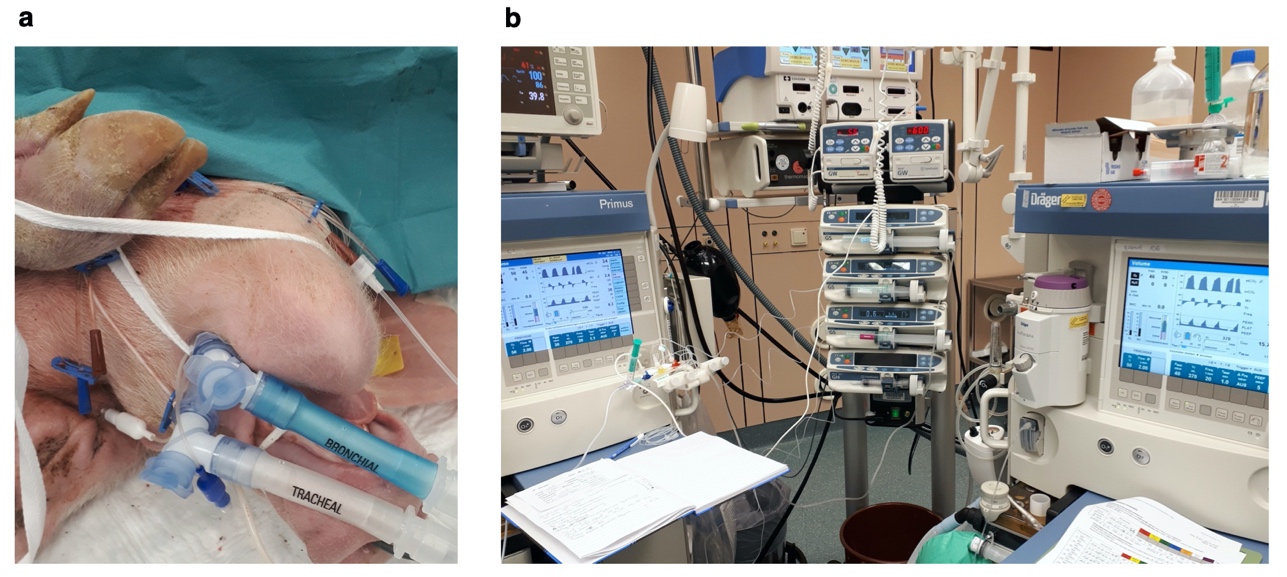


**Figure S2: Separation of ventilation of the right lung and the left lung**

(**a**) depicts the DLTm in situ with one ventilator connected to the tracheal lumen (white) and one ventilator connected to the bronchial lumen (blue). (**b**) depicts the ventilators performing ventilation of the left lung and ventilation of the right lung.


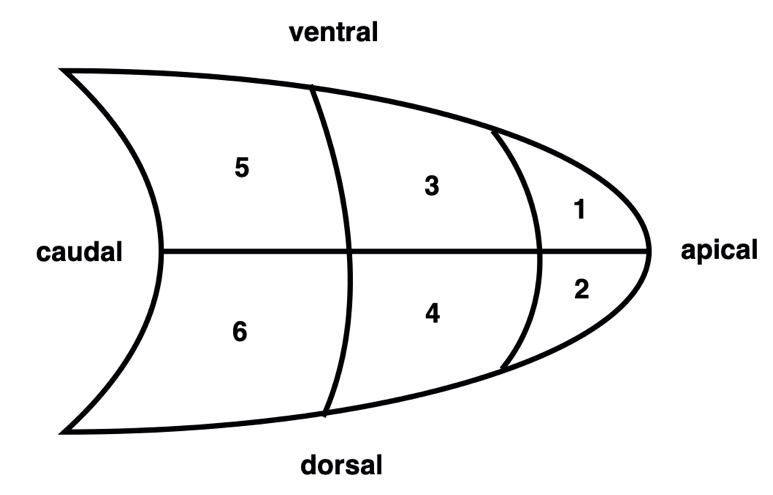


**Figure S3: Lung zones for histopathological analysis**

Schematic representation of the six zones of tissue sampling after the experiment; upper lobe, mid field and lower lobe, each ventral and dorsal, respectively.


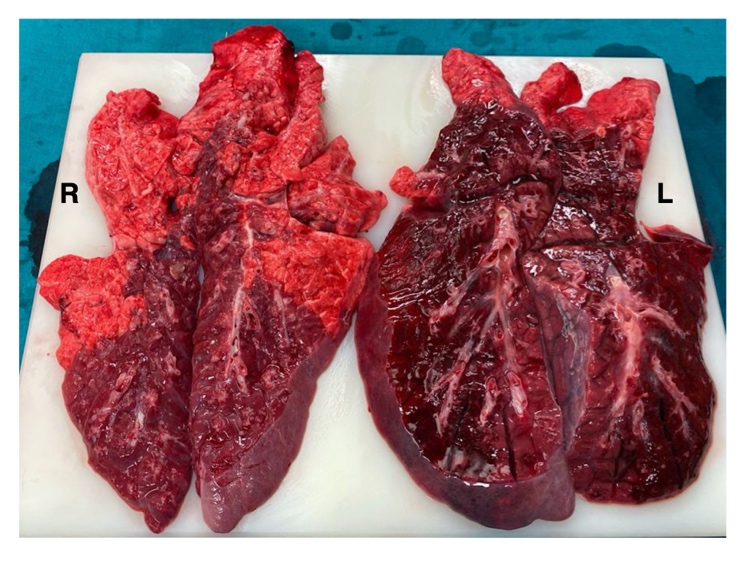


**Figure S4: Macroscopic preparation of a spill-over lung in the pilot study** Successful generation of ALI in the left lung (**L**) but clearly visible spill-over and injury in the lower lobe of the right lung (**R**). ALI induction occurred in supine position.

**Table S1:**

| **Table S1. Hemodynamics, metabolic, and oxygenation before and after ALI induction over time (main study)** | | | | | | | | | |
| --- | --- | --- | --- | --- | --- | --- | --- | --- | --- |
|  | Baseline | immediately  after ALI | 1h  after ALI | 2h  after ALI | 3h  after ALI | 4h  after ALI | 5h  after ALI | 6h  after ALI | 7h  after ALI |
| **Weight** | 61.7±7.2  (n = 6) | - | - | - | - | - | - | - | - |
| **FiO2**  **(%)** | 0.5±0.24  (n = 6) | - | - | - | - | - | - | - | - |
| **P/F ratio** | 371±96.6  (n = 6) | - | - | - | - | - | - | - | - |
| **PaO2**  **(mmHg)** | 194.5±135.5  (n = 6) | 141.3±48.1  (n = 6) | 134.7±74.9  (n = 3) | 178.6±134.1  (n = 3) | 138.4±59.4  (n = 3) | 188.5±142.3  (n = 4) | 185.2±156.9  (n = 3) | 213.2±117.5  (n = 3) | 87.3  (n = 1) |
| **PaCO2**  **(mmHg)** | 42.5±3.2  (n = 6) | 58.7±12.6  (n = 6) | 53.3±7.2  (n = 3) | 63.2±15.7  (n = 3) | 54.3±6.3  (n = 3) | 61.4±4.9  (n = 4) | 59.6±8.4  (n = 3) | 61.6±3.1  (n = 3) | 59.8  (n = 1) |
| **SaO2**  **(%)** | 99.5±0.8  (n = 6) | 98.7±1.6  (n = 6) | 97.3±2.5  (n = 3) | 97.7±2.1  (n = 3) | 97.3±3.8  (n = 3) | 96±5.2  (n = 4) | 98±2  (n = 3) | 99.3±1.2  (n = 3) | 98  (n = 1) |
| **Hemoglobin**  **(g/dL)** | 9.3±0.5  (n = 6) | 9.5±1.1  (n = 6) | 9.5±1.2  (n = 3) | 9.5±1.5  (n = 3) | 8.8±0.8  (n = 3) | 8±1.1  (n = 4) | 8.4±0.6  (n = 3) | 7.8±0.7  (n = 3) | 8.2  (n = 1) |
| **Lactate**  **(mmol/L)** | 1.2±0.3  (n = 6) | 2.2±1.5  (n = 6) | 2.7±2.7  (n = 3) | 2.9±1.3  (n = 3) | 3.3±1.6  (n = 3) | 3.2±1.2  (n = 4) | 2.5±1  (n = 3) | 1.4±0.3  (n = 3) | 1.2  (n = 1) |
| **BE**  **(mmol/L)** | 5.3±2  (n = 6) | 3.4±3.5  (n = 6) | 2.6±3.9  (n = 3) | 3.9±1.5  (n = 3) | 2.4±1.9  (n = 3) | 2.7±3.5  (n = 4) | 3.5±1.5  (n = 3) | 4.1±2.7  (n = 3) | 7.1  (n = 1) |
| **HR**  **(1/min)** | 85±5  (n = 3) | 123±18  (n = 6) | 124±10  (n = 4) | 126±12  (n = 4) | 135±13  (n = 4) | 139±18  (n = 4) | 141±15  (n = 4) | 128±9  (n = 4) | 141±18  (n = 4) |
| **MAP**  **(mmHg)** | 80±4  (n = 3) | 87±21  (n = 6) | 74±12  (n = 4) | 71±15  (n = 4) | 78±5  (n = 4) | 73±13  (n = 4) | 73±11  (n = 4) | 75±16  (n = 4) | 74±10  (n = 4) |
| Values are expressed as mean ± SD. P/F ratio = PaO2/FiO2; FiO2 = fraction of inspired oxygen; BE = base excess; HR = heart rate; MAP = mean arterial pressure | | | | | | | | | |

**Table S2:**

| **Table S2. Lung injury score (adapted from Matute-Bello et al.^17^)**  Score | | | |
| --- | --- | --- | --- |
| Parameter | 0 | 1 | 2 |
| A. Neutrophils in the alveolar space | none | 1-5 | >5 |
| B. Neutrophils in the interstitial space | none | 1-5 | >5 |
| C. Hyaline membranes | none | 1 | >1 |
| D. Haemorrhage | none/mild | moderate | severe |
| E. Edema | none/mild | moderate | severe |
| Score = ((20 x A) + (14 x B) + (7 x C) + (7 x D) + (2 x E)) / 100 | | | |
